# Highly improved light harvesting and photovoltaic performance in CdTe solar cell with functional designed 1D-photonic crystal via light management engineering

**DOI:** 10.1038/s41598-022-15078-w

**Published:** 2022-07-04

**Authors:** Çağlar Çetinkaya, Erman Çokduygulular, Barış Kınacı, Feyza Güzelçimen, Yunus Özen, Nihan Akın Sönmez, Süleyman Özçelik

**Affiliations:** 1 Physics Department, Faculty of Science, Istanbul University, 34134 Istanbul, Turkey; 2 Department of Engineering Sciences, Faculty of Engineering, Istanbul University-Cerrahpaşa, 34320 Istanbul, Turkey; 3 Department of Physics, Faculty of Science, Gazi University, 06500 Ankara, Turkey; 4 Photonics Application and Research Center, Gazi University, 06500 Ankara, Turkey; 5 Department of Photonics, Faculty of Applied Sciences, Gazi University, 06500 Ankara, Turkey

**Keywords:** Solar cells, Photonic crystals, Solar cells, Solar energy and photovoltaic technology

## Abstract

Photonic-based functional designs and integrations for advanced optoelectronic devices are regarded as promising candidates considering the enhancement of efficiency and tunability. With the aim to improve photovoltaic performance by increasing photon harvesting, the study presents the prominent findings of experimental and theoretical comparison of optical and electrical evaluation integrating a functionally designed one-dimension photonic crystal (1D-PC) into CdTe solar cells. Since transparency of the CdS/CdTe heterojunction based solar cell (SC) is reduced by a photonic band gap formed by (MgF_2_/MoO_3_)^N^ 1D-PC; namely, re-harvesting is improved by increasing absorbance. The period number at resonance wavelength of 850 nm and photocurrent density (${J}_{ph}$) have remarkable influence on the investigation. For four periods, the reflectance in the region of photonic band gap is sufficient for photon harvesting and saturation occurs. The photovoltaic performances are comparatively analysed for SCs with and without 1D-PC produced at optimal values. The open-circuit voltage does not change, besides, short-circuit current density and maximum-current density vary between 15.86–17.23 mA cm^−2^ and, 13.08–15.41 mA cm^−2^. Having integrated the 1D-PC into the structure, it is concluded that the FF and power conversion efficiency increase from 55.27 to 63.35% with an improvement of 15.91% and, from 8.26 to 10.47% with an improvement of 21.10%.

## Introduction

In today's world, where the increasing energy demand is high, the importance of the energy need for abundant, low-cost, and clean energy sources is increasing day by day. Therefore, renewable energy, an alternative to fossil fuel-containing sources, offers a solution to overcome this energy need. In solar cells (SC), which are the most promising alternative among renewable energy sources, photon energy from the sun can be collected cheaply, efficiently, and simply^1^. Especially Cadmium Tellurium (CdTe) based solar cells have recently attracted attention in academic and industrial studies due to their ability to reach 22.1% efficiencies^2,3^, high thermal cell stability, low cost, and long-term stable photovoltaic performance^4–7^.

CdTe has a direct optical band gap around 1.45 eV, which is the convenient band gap for SC’s. Also, due to the high absorbency of CdTe, CdTe-based SCs generally consist of a thin n-type Cadmium Sulfide (CdS) and a relatively thick p-type CdTe heterojunction. The efficiency of CdS/CdTe heterojunction SCs has been increased by 1.5% in the last 20 years under laboratory conditions, with cell efficiency exceeding 20% and module efficiency close to 15%^8–10^. Through the absorber CdTe, which provides high absorption at the joint, all photons with energies greater than the band gap can be absorbed even with a very thin p-type material. SCs based on CdS/CdTe heterojunctions with a CdTe absorber layer about 2 μm thick can absorb almost all photons from AM 1.5G. In addition, the flexibility of the CdS/CdTe heterojunction at this thickness is quite high. For this reason, the use of CdS/CdTe-based SCs in technology is very advantageous in terms of production, mechanical functionality, and lightness. The n-type CdS layer is a suitable thin window layer in the heterojunction and can form a suitable heterojunction with p-type CdTe. In cases where thin CdS films produce higher short-circuit current densities, it affects the cell's conversion efficiency^11^. In addition to the advantages, there are fundamental and significant challenges for high-efficiency CdS/CdTe-based systems for long-term thermal and mechanical stability. The most important of these is the compatibility of p-type CdTe with the bottom contact metal, which has a stable, non-rectifying, and low-resistance configuration. Metals used as bottom contact metals can diffuse into the cell over time, and this causes a critical decrease in cell efficiency. Therefore, after initial generation, systems based on heterojunction CdS/CdTe exhibit good photovoltaic performance, but the efficiency decreases over time. Therefore, this problem can be overcome by adding a Back Surface Area (BSF) with a high band gap material between the absorbent layer CdTe and the metal contact^12^. BSF also limits the movement of minority carriers around the active region and improves photovoltaic performance.

Even if the efficiency is high in SC consisting of CdTe/CdS heterojunction, a significant increase in efficiency has not been observed in recent years. Therefore, using multiple absorbers with different band gaps in tandem or a bifacial illuminated configuration may be viable to increase sunlight utilization^13^. As a result of the low absorption of CdTe acting as the absorber layer in the long-wavelength region of air mass 1.5 global (AM 1.5G) distribution, studies on appropriate band alignment have been carried out with band engineering to increase the absorption in the long-wavelength region^4,14–18^. Apart from the modifications to be made with different material alloying, doping, or band alignment in CdS and CdTe, light management engineering-based approaches can be tried to adjust the optical properties of the cell by modifying the propagation of the electromagnetic wave inside the cell without making any modifications directly to the CdS/CdTe heterojunction^19–22^. Among these approaches, the integration of a periodic one-dimension (1D) dielectric mirror (DM) layer with photonic crystal (PC) properties into the structure for improved and modifiable optical properties is an effective approach^20,23^. With this feature, 1D-PC systems are also included in the literature as Distributed Bragg Reflector (DBR) and are used functionally to increase the photovoltaic performance consisting of various material systems^21,24–26^. In the SC, which consists of 1D-PC and CdS/CdTe heterojunction, the unabsorbed photons—especially those with long wavelengths—can be sent back to the heterojunction by creating a suitable reflection, thus increasing the absorption.

1D-PCs consist of structures with two or more dielectric constants grown or deposited in a single direction, and therefore the dielectric constant and refractive index periodically change in one direction in these structures^27^. Thence, with a suitable 1D-PC design, a photonic band gap formed in the wavelength range where reflection will occur for the propagation of photons in a single direction can be created. By optimizing the thickness and number of layers of 1D-PC system, the width and reflection intensity of the photonic band gap can be adjusted. Thus, the absorption characteristics can be modified by especially adjusting the reflection characteristics without changing the properties of the active layer as a result of the integration of 1D-PCs in CdS/CdTe SC. By sending the photons that are not absorbed in the CdS/CdTe heterojunction to the active region by internal reflection, the photocurrent density (${J}_{ph}$) in the SC can be increased. Thus, the photovoltaic performance of the cell can be improved.

In the literature, there are studies in which an increase in photovoltaic performance is observed with the integration of Si/SiO_2_ DBR system into CdS/CdTe-based SCs^9,26^. However, in these studies, physical interpretation was made only on the optical spectra of the DBR layers and not on the optical properties of the SC. In addition, the bandwidths are kept quite wide in the related studies. Because in the Si/SiO_2_ DBR system, the refractive index contrast is high, and a wide photonic band gap is obtained. So it is not possible for SCs to work with bifacial illumination. For bifacial working condition, it should be aimed that the reflection band is not wide for both top and bottom illumination, that is, material systems with low refractive index contrast.

In the present study, we focused on an effective photonic band gap design for only the wavelength region with low absorption, based on the optical properties of each layer in the CdS/CdTe SC and 1D-PC integration. The focus is on improving the photovoltaic performance by integrating the appropriate design N period (MgF_2_/MoO_3_)^N^ 1D-PC into the SC with CdS/CdTe heterojunction. For this purpose, the optical properties of CdS/CdTe heterojunction SC were investigated in detail, both experimentally and theoretically, and it was integrated into the (MgF_2_/MoO_3_)^N^ 1D-PC SC with a functionally fine-tuned photonic band gap property to improve absorption by photon harvesting. In the production of SCs, RF sputter technique was used for the deposition of materials. Methodologically, we designed photonic band gap in the near-infrared region (NIR) and in different periods for the transparent wavelength region in the absorption band of CdTe, which is the absorber layer. We calculated the optical characteristics of the designed (MgF_2_/MoO_3_)^N^ 1D-PCs and the SCs formed by integrating these 1D-PCs into the SC with CdS/CdTe heterojunction using the Transfer Matrix Method (TMM). We produced CdTe-based SCs with and without 1D-PC, whose optimal period was determined based on the theoretically calculated ${J}_{ph}$, and we presented the photovoltaic performance comparatively. We observed a significant increase in photovoltaic performance by improving the NIR absorption characteristic of 1D-PC and CdTe-based SCs.

## Results

**Figure 1:**
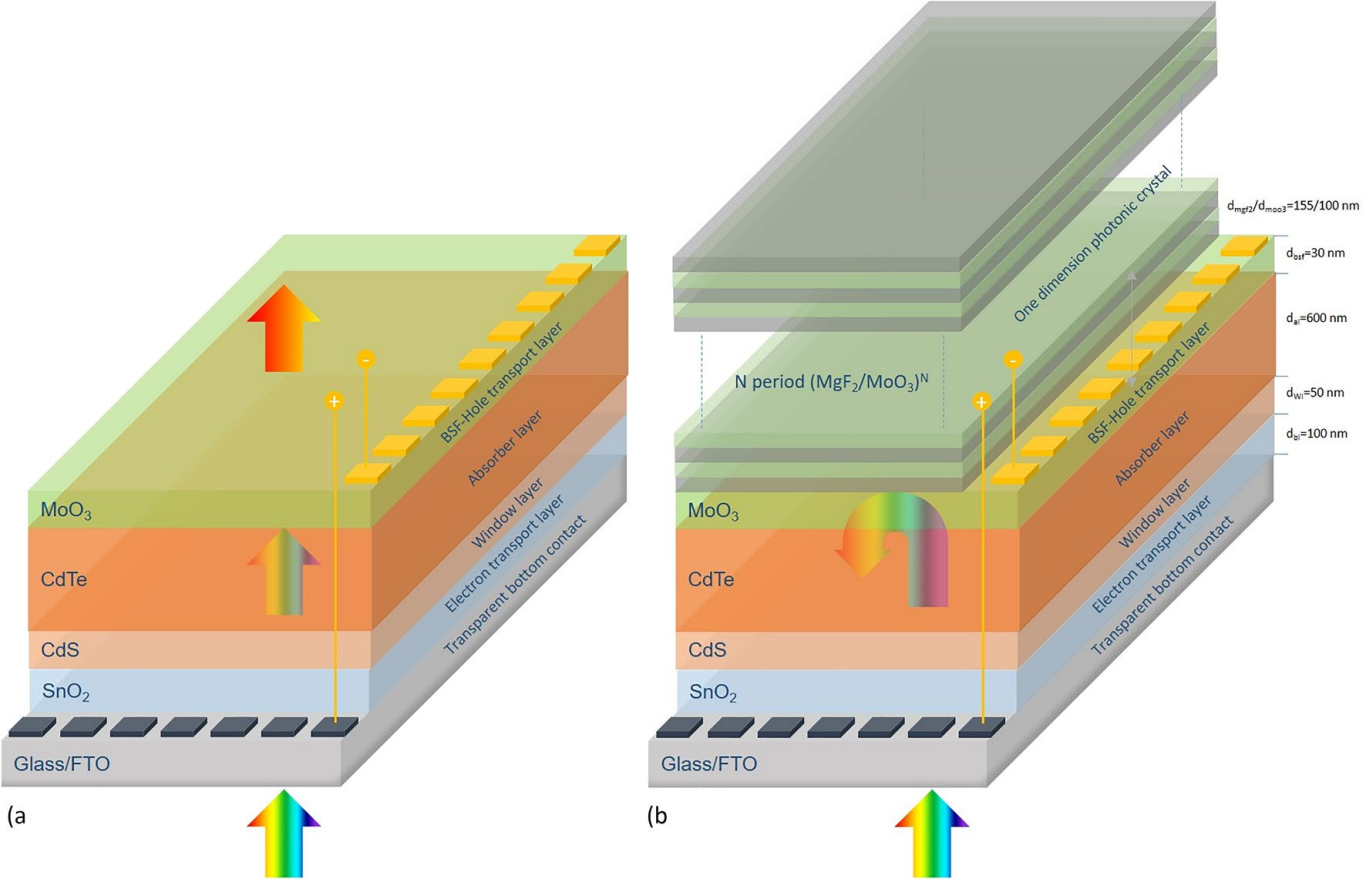
CdTe-based SCs: (**a**) FTO/SnO_2_/CdS/CdTe/MoO_3_ and (**b**) FTO/SnO_2_/CdS/CdTe/MoO_3_/(MgF_2_/MoO_3_)^N^.

In the study, the changes in photovoltaic performance result from the integration of the (MgF_2_/MoO_3_)^N^ 1D-PC system designed inappropriate parameters based on the optical properties of the SC formed in the FTO/SnO_2_/CdS/CdTe/MoO_3_ SC were investigated. The investigated FTO/SnO_2_/CdS/CdTe/MoO_3_ and FTO/SnO_2_/CdS/CdTe/MoO_3_/(MgF_2_/MoO_3_)^N^ SCs are given in Fig. 1a,b, respectively. Fluorine Tin Oxide (FTO) coated on glass substrate was used as the bottom contact in the produced SCs. FTO is electrically conductive and optically highly transparent in the wavelength range for which AM 1.5G is responsible. 100 nm thick SnO_2_ was used on FTO due to its wide optical band gap, high optical and electrical properties to prevent recombination of photogenerated carriers. At this thickness, SnO_2_ is transparent and at the same time, it provides band arrangement and prevents the leaking of the holes formed by photoproduction to the bottom contact. In this respect, SnO_2_ also acts as an electron transport layer (ETL). A 50 nm thick CdS window layer is added to the structure to absorb electromagnetic waves and transmit photogenerated electrons to the SnO_2_ layer. CdS is a promising n-type material from group II-VI and has a wide direct band gap (2.42 eV)^28,29^. In order to increase the photovoltaic performance by creating a large number of electron–hole pairs, CdTe material with an optical band gap of 1.5 eV was chosen as the active layer in the structures produced. CdTe with high material quality can be achieved with the RF sputter technique, especially for submicron thicknesses^30^. In addition to the majority carriers formed as a result of photogeneration, 100 nm thick MoO_3_ is used as the BSF layer to localize the minority carriers around the pn junction to collect them more efficiently under the effect of the internal electric field and to reduce recombination. Due to its convenient work function and high p-type doping capability, the formation of ohmic contacts with MoO_3_ is both easier and selective transport is provided by preventing photogenerated electrons from reaching the top contact in the structure^13^. In this respect, MoO_3_ also acts as a hole transport layer (HTL) in SC. Also, in our previous studies, we have structural and morphological investigations for MoO_3_ thin film^31,32^. In order to improve the absorption by reducing the transparency in the FTO/SnO_2_/CdS/CdTe/MoO_3_ SC, the photonic band gap in the structure is designed with 1D-PC, which consist of MgF_2_ and MoO_3_ have different dielectric constants and therefore different refractive indices.

The materials excluding the metallization parts were deposited using the RF sputter technique in the SCs examined in the study. The RF sputter technique provides homogeneous and thickness-controlled deposition for CdTe, CdS, and other metal-oxide alloys at the desired stoichiometric ratio^19,30,31,33–35^. In addition, in our previous study, we found that the optical calculations we made with TMM on the devices we produced by deposition with the RF sputter technique showed a nearly perfect agreement with the experiment^19^.

**Figure 2:**
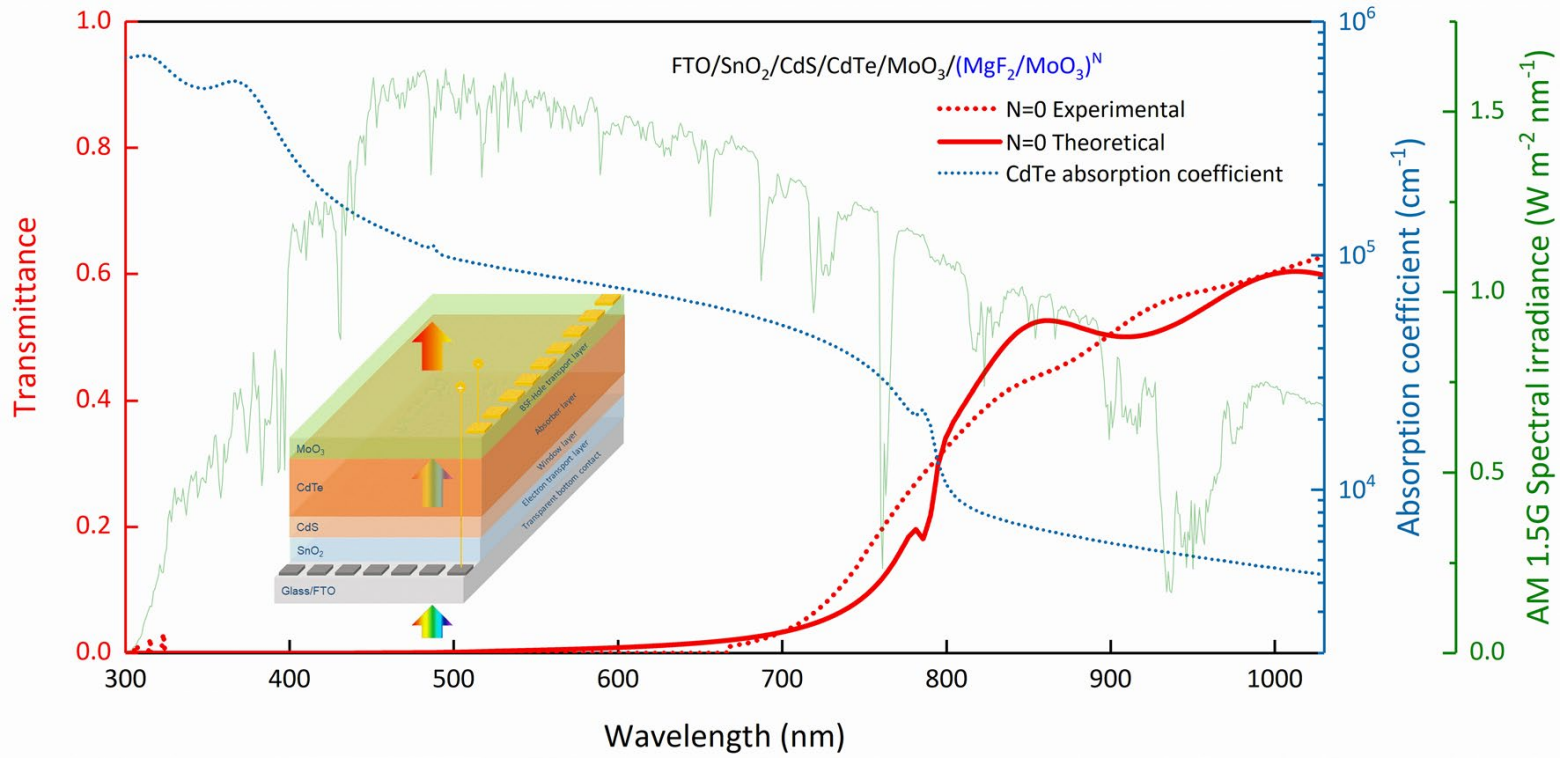
Experimental and TMM calculated transmittance spectra of FTO/SnO_2_/CdS/CdTe/MoO_3_/(MgF_2_/MoO_3_)^N^ SC for N = 0, change of absorption coefficient of CdTe and AM 1.5G spectral irradiance depending on wavelength. 1D-PC-free SC is given in the inset.

In the examined SCs, the absorber layer is CdTe, which is relatively thicker and has a higher absorption coefficient than the other layers. Therefore, to achieve an increase in photovoltaic performance as a result of only 1D-PC integration without making any modifications to the heterojunction or the SC, first of all, the optical characteristic of the entire SC should be known. Therefore, we started the investigation based on the experimental and calculated transmittance spectra with TMM for the 1D-PC-free SC (FTO/SnO_2_/CdS/CdTe/MoO_3_). Since the transmittance of the SC will increase in the region where the absorption decreases, we first focused on the longer wavelength region –NIR–, starting from about 700 nm, which is the wavelength region where the transmittance starts and increases. In Fig. 2, the SC's experimental and calculated transmittance spectra, the variation of the absorption coefficient of CdTe and AM 1.5G spectral irradiance depending on the wavelength are given.

The fact that the trends are very close depending on the wavelength in the experimental and theoretical transmittance spectra calculated with TMM in Fig. 2 shows that TMM is a powerful method. CdTe-based SC’s were deposited by RF sputter technique and the layers may be deposited with imperfect flatness and slight deviations from the desired thicknesses in terms of nm. This small difference may have occurred because the calculations made with TMM were calculated for the ideal condition of the layers with perfect flatness over the thicknesses in terms of nm. Accordingly, a slight difference is observed in some wavelength regions. The absorption coefficient of the absorber layer CdTe decreases, especially after 800 nm. However, the absorption characteristic is still observed in the NIR region, albeit slightly. The fact that the transparency of the FTO/SnO_2_/CdS/CdTe/MoO_3_ SC also increases after 700 means that photons with wavelengths longer than 700 nm pass through the SC without being absorbed. Increasing the absorption by reducing the transparency in this region, i.e., reflecting unabsorbed photons back into the active region and obtaining an efficient harvest, can positively affect the photovoltaic performance of the cell.

**Figure 3:**
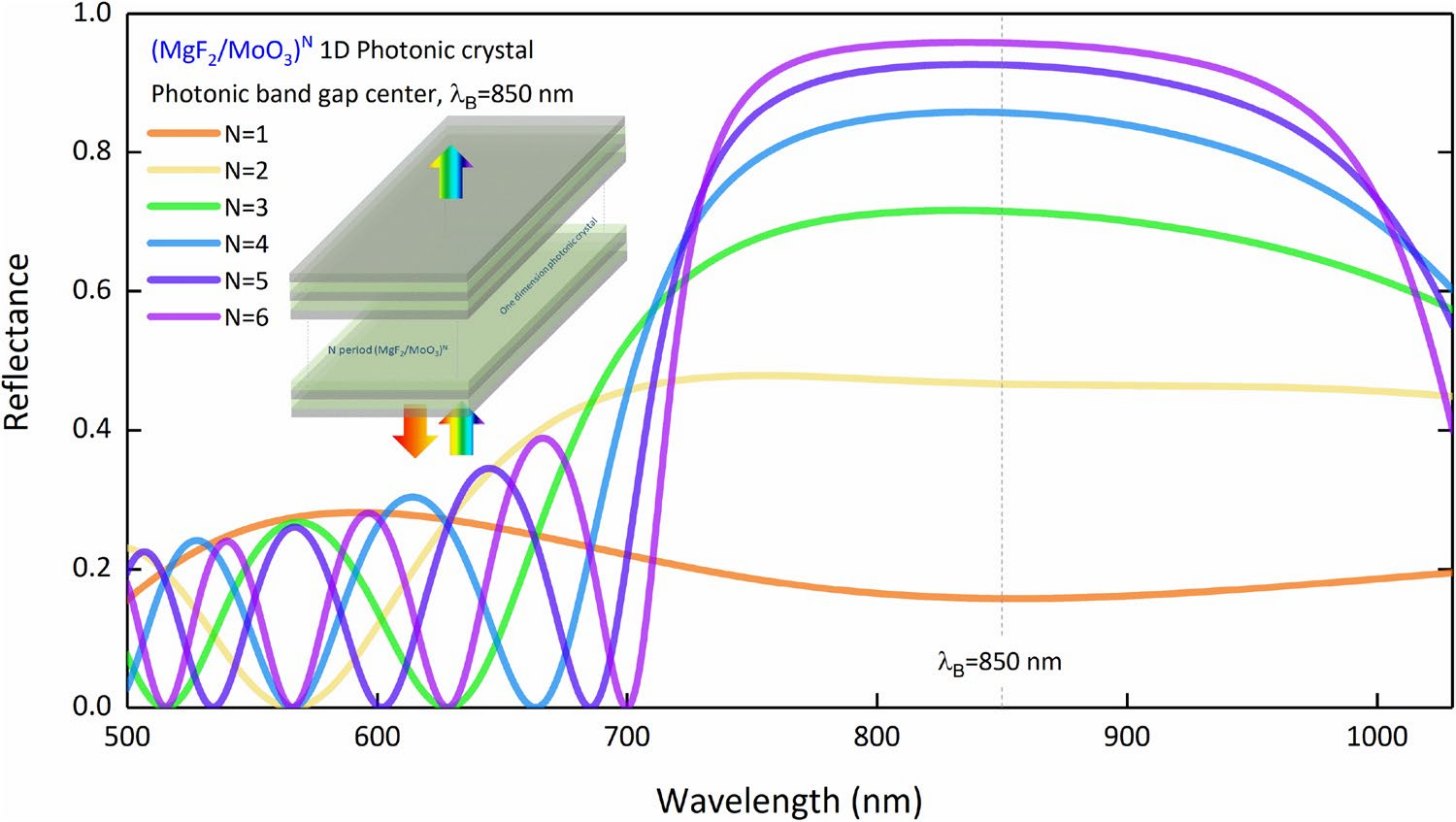
Reflection spectra of (MgF_2_/MoO_3_)^N^ 1D-PC system at different periods for ${\lambda }_{B}$=850 nm. 1D-PC system is given as inset.

In studies on DBR integration into CdS/CdTe-based SCs, only physical interpretation was made on the optical spectra of the DBR layers, and no evaluation was made on the optical properties of the entire SC^9,26^. In addition, the bandwidths were kept quite wide in the related studies and therefore, it was not possible for SCs to work with bifacial illumination. In principle, the refractive indices of the materials that make up the photonic crystal and the contrast of these refractive indices determine the characteristics of the photonic band gap or stop-band to be created with DBR. Increasing the refractive index contrast between materials increases both the reflection intensity and the photonic bandwidth. Therefore, a narrower photonic band gap can be obtained with materials whose refractive indices are closer to each other (less contrast) for a given central wavelength. We offer a methodologically more effective CdS/CdTe SC design that can be improved in the wavelength region where the absorption is low and can operate under bifacial illumination. For this purpose, we try to reduce the permeability of the with a 1D-PC design with photonic band gap only in the NIR region. Therefore, we calculated the reflectance spectra of the (MgF_2_/MoO_3_)^N^ 1D-PC system with a central wavelength of ${\lambda }_{B}$=850 nm at different periods by TMM (Fig. 3). For ${\lambda }_{B}$=850 nm, the thicknesses of MgF_2_ and MoO_3_ are 155 and 100 nm, respectively. When Fig. 3 is examined, 1D-PC is designed to act as a mirror in the desired wavelength range according to the mentioned methodology.

**Figure 4:**
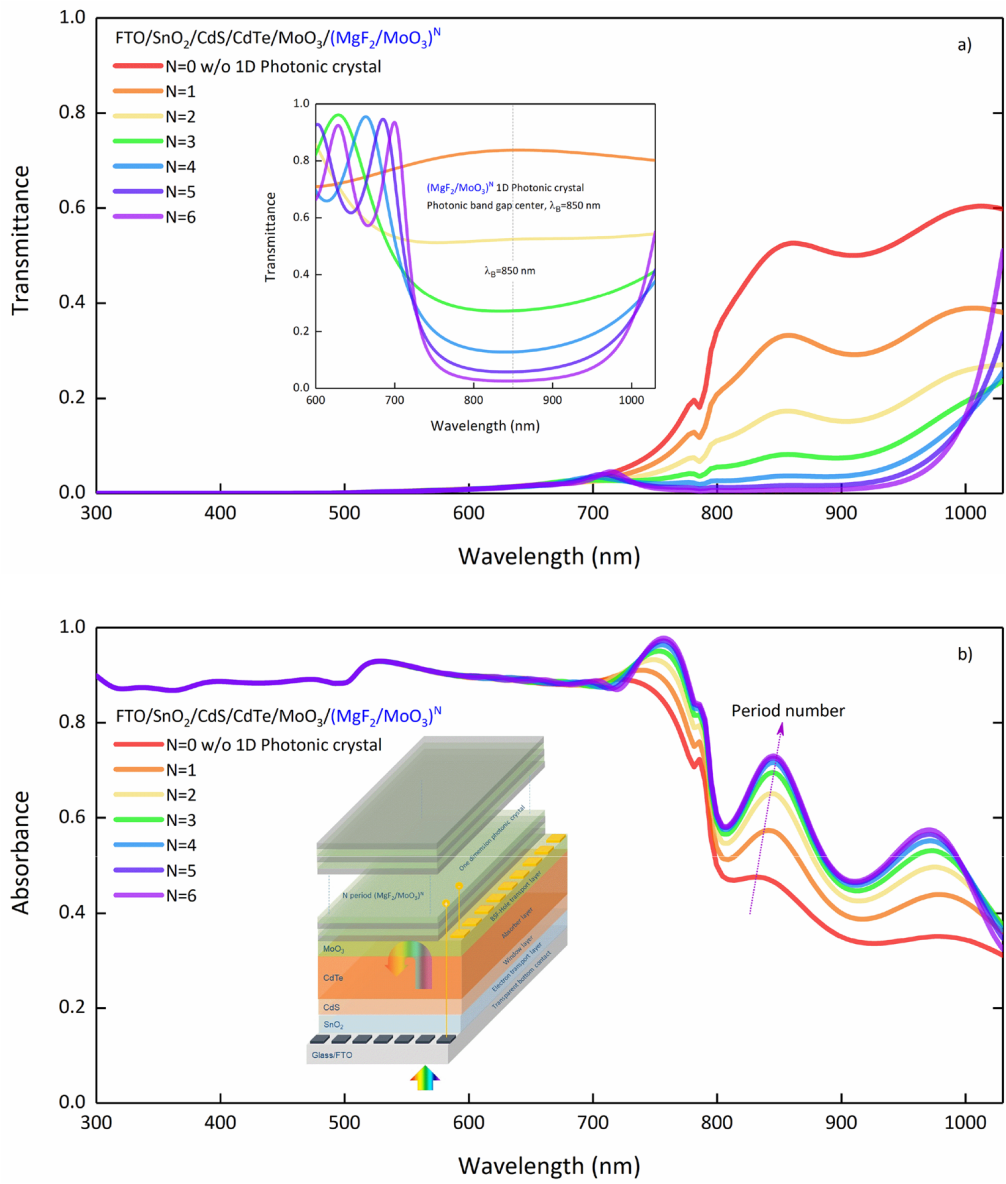
(**a**) Transmittance and (**b**) absorbance spectra of the FTO/SnO_2_/CdS/CdTe/MoO_3_/(MgF_2_/MoO_3_)^N^ SC calculated for different periods at ${\lambda }_{B}$=850 nm. The transmittance spectrum of (MgF_2_/MoO_3_)^N^ 1D-PC as inset is given in (**a**). In (**b**), the SC containing 1D-PC is given as inset.

The (MoO_3_/MgF_2_)^N^ 1D-PC system’s refractive index contrast is not much, and thus a narrower photonic band gap can be obtained. Increasing the number of periods in the (MgF_2_/MoO_3_)^N^ 1D-PC system narrows the photonic bandwidth and increases reflection intensity. The rate of increase in the reflection intensity decreases as the number of periods increases. For N = 5 and 6 periods, accumulation starts at 90% reflectance. As expected, the period does not affect the center wavelength. In addition, the short wavelength tail of the photonic band gap formed by the (MgF_2_/MoO_3_)^N^ 1D-PC system shifts from 657 to 700 nm as the period increases from 1 to 6. Therefore, by integrating the (MgF_2_/MoO_3_)^N^ 1D-PC system into the FTO/SnO_2_/CdS/CdTe/MoO_3_ SC, it is necessary to examine how it affects the increasing SC transparency after 700 nm. The transmittance and absorption spectra were calculated for different periods of the FTO/SnO_2_/CdS/CdTe/MoO_3_/(MgF_2_/MoO_3_)^N^ SC at ${\lambda }_{B}$=850 nm are given in Fig. 4a,b, respectively.

As intended, the transmittance spectrum of the FTO/SnO_2_/CdS/CdTe/MoO_3_/(MgF_2_/MoO_3_)^N^ SC was decreased with the increase of the period number after 700 nm with the (MgF_2_/MoO_3_)^N^ 1D-PC. Increasing the number of periods up to N = 4 in the SC reduces the transmittance up to 1000 nm to almost zero. At around 700 nm, the intersection of the transparency of the FTO/SnO_2_/CdS/CdTe/MoO_3_ SC and the transparency of the (MgF_2_/MoO_3_)^N^ 1D-PC (Fig. 4a inset) is evident. Therefore, the (MgF_2_/MoO_3_)^N^ 1D-PC system designed for ${\lambda }_{B}$=850 nm is a suitable design for CdTe-based SC. In high periods (N > 4), the photonic band gap having sharp lines and decreasing width tends to increase the transmittance around 1000 nm. In order to interpret the effect of this critical change in the optical characteristic of the SC on the photovoltaic performance, it is necessary to examine the absorbance spectrum.

**Figure 5:**
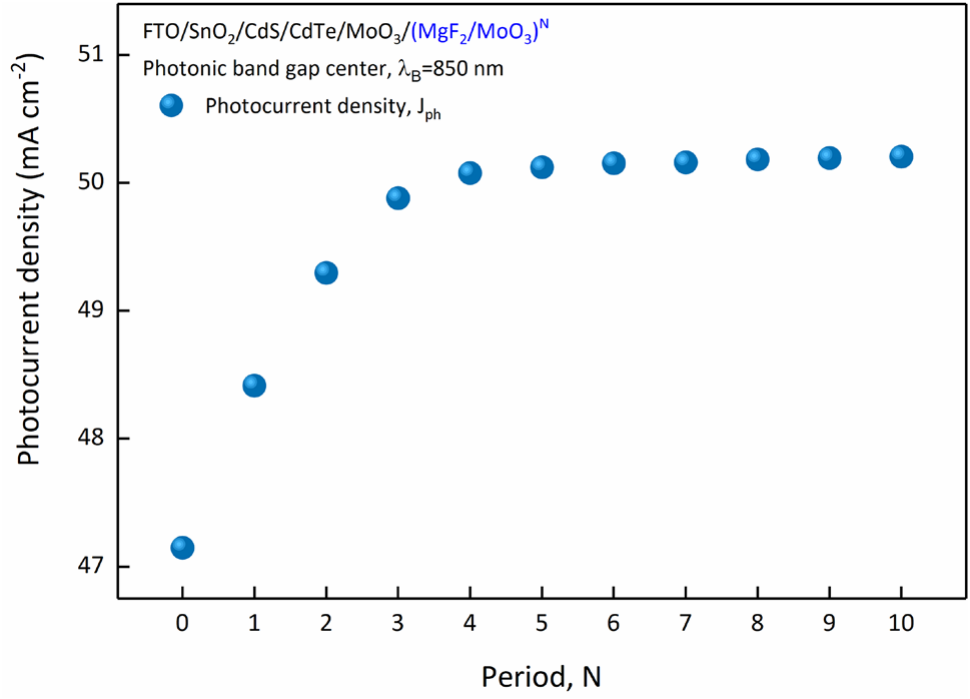
Variation of ${J}_{ph}$ according to the number of periods in FTO/SnO_2_/CdS/CdTe/MoO_3_/(MgF_2_/MoO_3_)^N^ SC.

In the FTO/SnO_2_/CdS/CdTe/MoO_3_/(MgF_2_/MoO_3_)^N^ SC, decreasing the transmittance after 700 nm with the period number directly affects the absorption spectrum, and an increase in absorption was observed after 700 nm. Similar to the trend in the transmittance spectrum, there is no significant increase in absorption in the NIR region with a period number greater than N = 4. In order to examine how this improvement in absorption with 1D-PC in CdTe-based SCs affects photovoltaic performance, AM 1.5G spectral irradiance distribution should also be examined. Therefore, for a more effective evaluation, we calculated the absorption characteristic of SC and ${J}_{ph}$ over ${S}_{AM1.5G}$ using Eq. (16). Here, when calculating the ${J}_{ph}$, it is assumed that each photon creates an electron and a hole in the SC^19^. This situation provides a relative evaluation and allows us to understand whether the flow mechanisms in the SC have improved relatively or not. The variation of ${J}_{ph}$ in FTO/SnO_2_/CdS/CdTe/MoO_3_/(MgF_2_/MoO_3_)^N^ SC according to the number of periods is given in Fig. 5.

${J}_{ph}$ increases significantly in SC up to N = 4 periods and becomes saturated for higher periods. Increasing the reflection in (MgF_2_/MoO_3_)^N^ 1D-PC up to N = 4 periods significantly reduces the transmittance of SC, especially by sending photons with wavelengths greater than 700 nm back into the active region. The improvement in absorption by sending photons that were not absorbed in the active region into back with 1D-PC led to re-harvest and increased ${J}_{ph}$. Therefore, the integration of (MgF_2_/MoO_3_)^N^ 1D-PC produced in 4 periods into CdTe-based SC is sufficient for the required re-harvesting of photons. This examination shows that the structural parameters of 1D-PC can be determined experimentally with TMM and an effective methodology without excessive material consumption and fabrication process.

**Figure 6:**
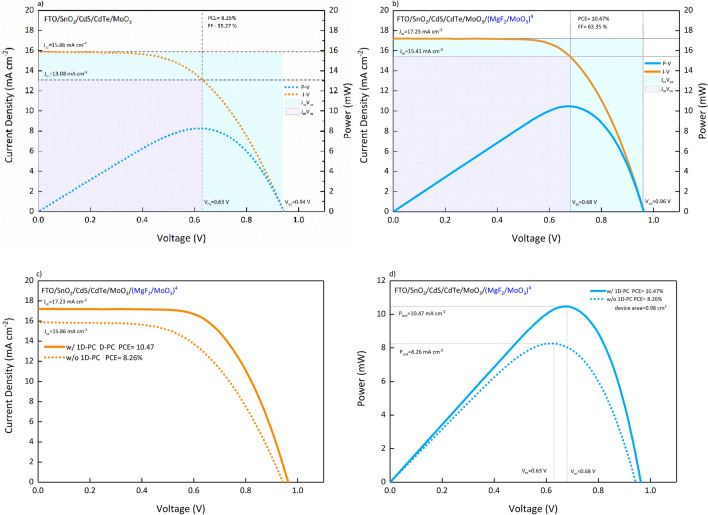
$J-V$ and P$-V$ characteristics of (**a**) FTO/SnO_2_/CdS/CdTe/MoO_3_ and (**b**) FTO/SnO_2_/CdS/CdTe/MoO_3_/(MgF_2_/MoO_3_)^4^ SCs. In (**c**,**d**), $J-V$ and $P-V$ characteristics of SCs are presented, respectively.

In order to determine how the improvement was brought about by the (MgF_2_/MoO_3_)^N^ 1D-PC system in ${J}_{ph}$ affects the photovoltaic performance of the cell and the cell output parameters, we produced the FTO/SnO_2_/CdS/CdTe/MoO_3_/(MgF_2_/MoO_3_)^4^ SC, which we determined as the optimal N = 4 period. We fabricated SCs with and without 1D-PC at the same parameters and deposition conditions to compare their photovoltaic performances for a quantitative comparison. The $J-V$ and $P-V$ characteristics of FTO/SnO_2_/CdS/CdTe/MoO_3_ and FTO/SnO_2_/CdS/CdTe/MoO_3_/(MgF_2_/MoO_3_)^4^ SC are given comparatively in Fig. 6a,b, respectively. Photovoltaic performance outputs obtained and calculated from $J-V$ and $P-V$ characteristics are given in Table 1.

**Table 1:** Photovoltaic performance parameters of FTO/SnO_2_/CdS/CdTe/MoO_3_ and FTO/SnO_2_/CdS/CdTe/MoO_3_/(MgF_2_/MoO_3_)^4^ SCs.

CdTe-based SC	${J}_{sc}$(mA cm^−2^)	${J}_{m}$(mA cm^−2^)	${J}_{ph}$(mA cm^−2^)	*V*_*m*_ (V)	*V*_*oc*_ (V)	*FF* (%)	*PCE* (%)
w/o 1D-PC	15.86	13.08	47.15	0.63	0.94	55.27	8.26
w/ 1D-PC	17.23	15.41	50.02	0.68	0.96	63.35	10.47

Integration of the (MgF_2_/MoO_3_)^4^ 1D-PC system into CdTe-based SC does not cause a significant change in ${V}_{oc}$. Because the top contact Au is located on MoO_3_ acting as BSF, and therefore the 1D-PC integration does not change the band arrangement of the SC. (MgF_2_/MoO_3_)^4^ 1D-PC integration improved ${J}_{sc}$ from 15.86 to 17.23 mA cm^−2^ and ${J}_{m}$ from 13.08 to 15.41 mA cm^−2^. This increase in current densities without any change in ${V}_{oc}$ shows that with the integration of the (MgF_2_/MoO_3_)^4^ 1D-PC system into the CdTe-based SC, the absorption is improved without any electrical change. This change is in good agreement with the calculated ${J}_{ph}$.

When the $J-V$ and $P-V$ characteristics presented in Fig. 6c,d are examined comparatively, the fact that the (MgF_2_/MoO_3_)^4^ 1D-PC system has increased the current densities as well as the ${V}_{m}$ has made the $J-V$ curve more diagonal, making the $FF$ 15.91% rate, it improved from 55.27 to 63.35%. In addition, the observed increase in current densities increased the maximum power obtained from the SC by integrating the (MgF_2_/MoO_3_)^4^ 1D-PC system into the CdTe-based SC, increasing the $PCE$ from 8.26 to 10.47%, increasing by 21.10%.

**Figure 7:**
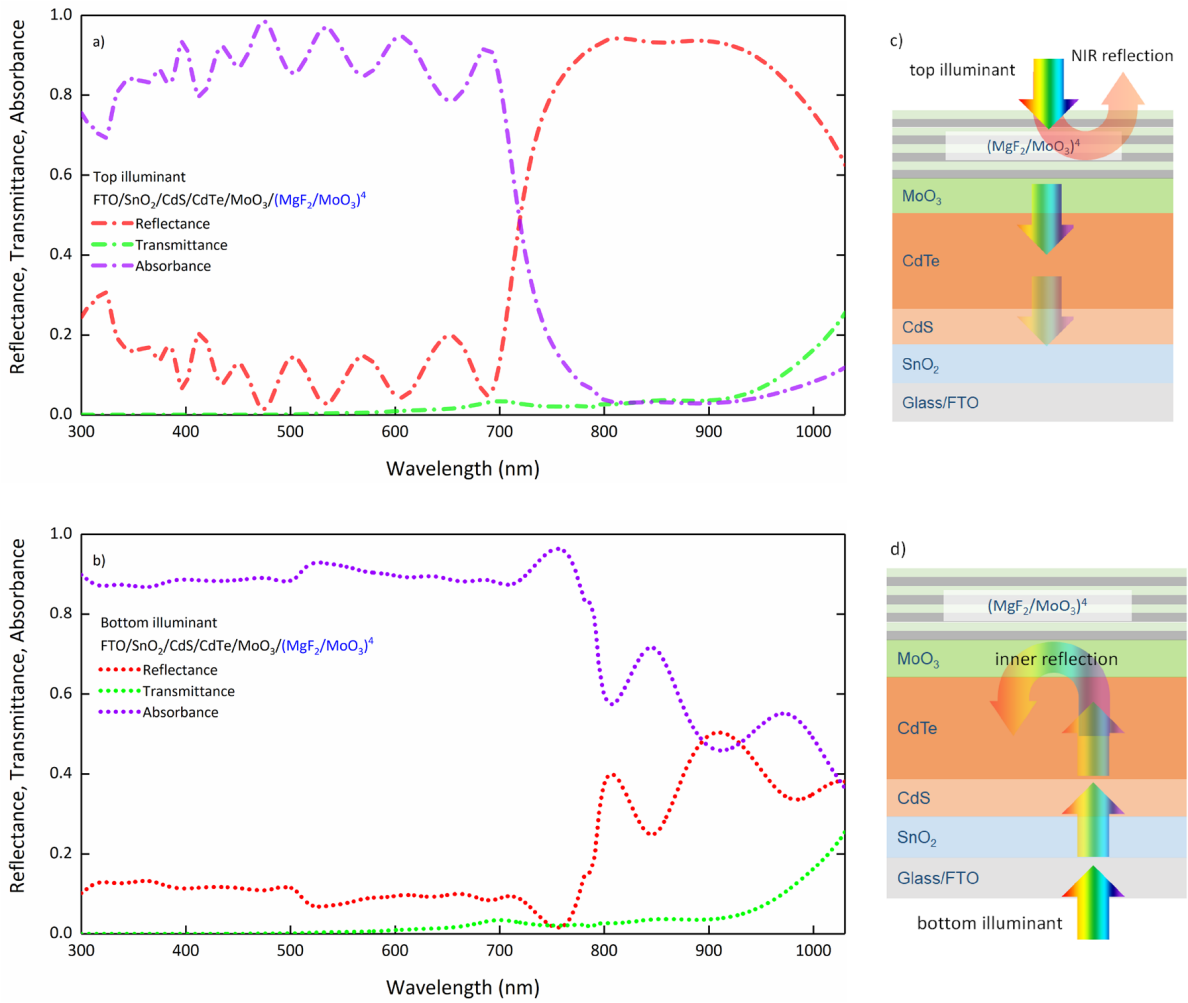
Optical characteristics of FTO/SnO_2_/CdS/CdTe/MoO_3_/(MgF_2_/MoO_3_)^4^ SC under (**a**) top and (**b**) bottom illumination, (**a**) representation of the optical processes occurring in the SC under (**c**) top and bottom illumination.

For FTO/SnO_2_/CdS/CdTe/MoO_3_/(MgF_2_/MoO_3_)^4^ SCs that are optimally produced in N = 4 periods, the functional photonic band gap design and the reflection—improvement in absorption—created in the NIR region allow the SC to work under the top and bottom illumination, that is, on both sides. Optical spectra of FTO/SnO_2_/CdS/CdTe/MoO_3_/(MgF_2_/MoO_3_)^4^ SC under the top and bottom illumination are given in Fig. 7. It is seen that in the case of top illumination, only 700 nm and longer wavelengths at the wavelengths for which AM 1.5G is responsible are not directly reflected by (MgF_2_/MoO_3_)^4^ 1D-PC and inserted into the SC. However, the absorption in other regions is quite high.

The fact that MoO_3_ with a thickness of 40 nm, which acts as the BSF layer, is very transparent in the visible and IR region allows photons of wavelengths not reflected by the photonic band gap of 1D-PC to enter the active region under top illumination. Therefore, the presence of SnO_2_ on the bottom side of the SC and MoO_3_ on the top side provides symmetry for the optical path that light will follow in the SC, since the optical characteristics of these materials are the same, especially for the IR region. Therefore, the transmittance characteristic of the SC does not change under the top and bottom illumination, and the absorption-reflection is in exchange with each other. Modification for the optical trace is done with photonic band gap. This shows that the functional photonic band gap design targeted in the study is methodologically suitable and light management is provided for the SC to work bifacially. As mentioned above, photon harvesting is increased with internal reflection under low illumination, allowing both an improvement in photovoltaic performance and an efficient photon harvest under top illumination.

## Discussion

The study was focused on the increment of photon harvesting and improvement of photovoltaic performance with a modifying optical characteristic due to functionally designed 1D-PC integration into CdS/CdTe-based SCs. The absorbance of the SC is enhanced by reducing the transparency with an appropriate photonic band gap which corresponds to the long-wavelength part of the CdTe absorbance band. A photonic band gap was designed with (MgF_2_/MoO_3_)^N^ 1D-PC, and the optical characteristics of the CdTe-based SC were calculated by TMM. The optimal period number of the PC was determined based on the calculated photocurrent density. In the (MgF_2_/MoO_3_)^N^ 1D-PC system, which is functionally designed at 850 nm resonance wavelength, the number of periods supports the absorbance by reducing the transparency in the NIR region of the CdTe-based SC. For the period numbers higher than four, accumulation started around 90% reflection and saturation was observed in the ${J}_{ph}$. The small-wavelength tail of the photonic band gap formed by 1D-PC system shifted from 657 to 700 nm as the period increases to six. This variation range covered the exact intersection of the transmittance spectrum of the CdTe-based SC and the photonic band gap of the 1D-PC.

It has been determined that an optimally four period (MgF_2_/MoO_3_)^4^ 1D-PC system is sufficient to increase photon harvesting and improve photovoltaic performance in CdTe-based SCs. CdTe-based SCs with and without 1D-PCs at optimal values were produced under the same deposition conditions and their photovoltaic performances were presented comparatively. It was determined that the (MgF_2_/MoO_3_)^N^ 1D-PC system did not change the band arrangement in the CdTe-based SC and only affected the optical characteristics. As a result of 1D-PC integration, there was no significant change in ${V}_{oc}$, ${J}_{sc}$ improved from 15.86 to 17.23 mA cm^−2^ and ${J}_{m}$ from 13.08 to 15.41 mA cm^−2^. These increments in current densities indicated that photons absorbed were reflected into the active region, and, re-harvesting occurred thanks to the photonic band gap designed in NIR. Consequently, $FF$ was improved by 15.91% from 55.27 to 63.35%, and $PCE$ was increased by 21.10% from 8.26 to 10.47%, dependent on the functionally designed 1D-PC integration.

It remains essential to increase photon harvesting and efficiency with light management engineering in 1D-PC and SCs without different material alloying, doping or band alignment. Unlike the studies conducted in the literature for this purpose, designing a functional photonic band gap only in the wavelength region where photon harvesting is weak shows the originality and potential to be a pioneer for future studies of the present study. In addition, the photonic band gap in the study is fine-tuned and functional, as well as not having a conventional wide band gap, allowing light to enter the SC from the top side.

In an overview of the study, it is verified that for the bifacial CdS/CdTe design with 1D-PC, very efficient photon harvesting is provided from the bottom side. Furthermore, an appropriate absorbance characteristic is provided for sufficient photon harvesting from the top side.

## Material and methods

### Theoretical background

Optical characteristics such as reflection, absorption and transmittance of the (MgF_2_/MoO_3_)^N^ 1D-PC system and FTO/SnO_2_/CdTe/CdS/MoO_3_/(MgF_2_/MoO_3_)^N^ SC designed within the scope of the study were calculated using the Transfer Matrix Method (TMM), which is a very effective method used in the simulations of various multilayer optoelectronic devices. TMM is a method that examines the propagation of electromagnetic waves in the structure and theoretically determines the optical properties of the structure^36–38^. The electromagnetic wave's electric and magnetic field components are interconnected in each layer by a transfer matrix. The propagation field components in the layers are connected with the propagation matrix at the interface of each layer.

**Figure 8:**
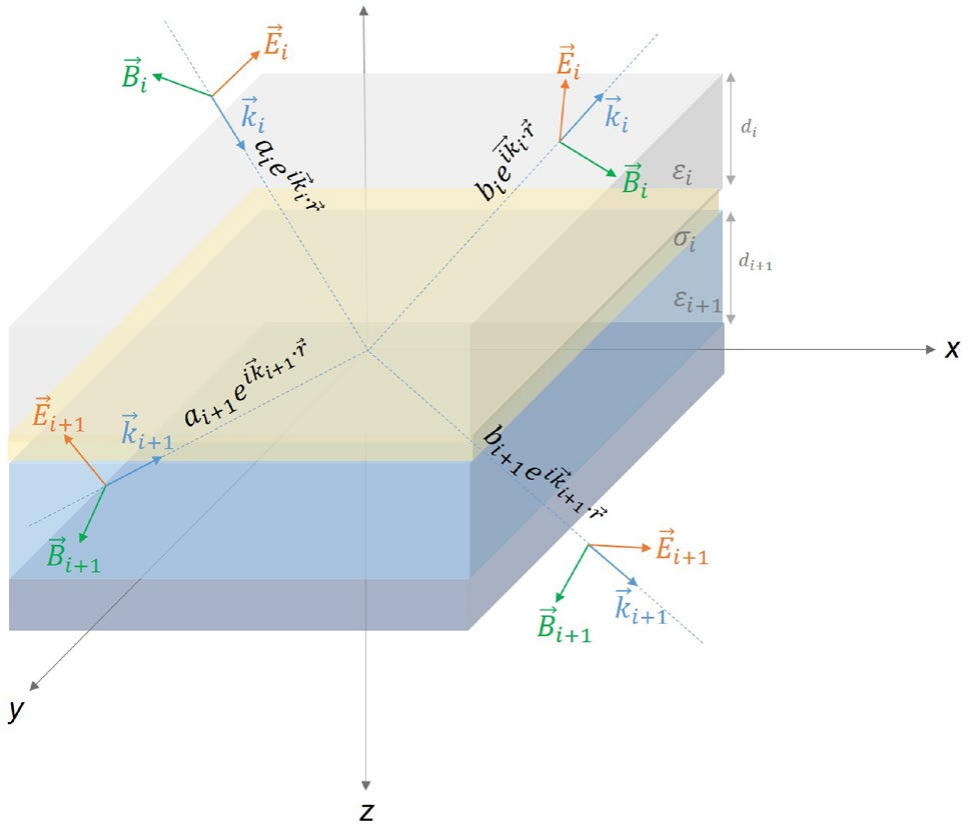
A structure consisting of dielectric layers with different ${\varepsilon }_{i}$.

With TMM, calculations can be made on structures designed by integrating metal layers with different conductivity and dielectric materials. In the most general case, this provides a general framework that allows the calculation of optical characteristics due to the integration of both dielectric, metal, and semiconductor materials with each other. Therefore, we took the most general form as a basis for explaining the calculations and performed the calculations by making the necessary reductions for the structures designed in the study. The plane of a metal layer sandwiched by two dielectrics can be taken as parallel to the $z$=0 planes. We can examine the propagation of electromagnetic waves on the interface of conductor and dielectric with the configuration and orientation in Fig. 8.

It can be thought that the electromagnetic wave is propagated in the $z$ direction and polarized in the $y$ direction by choosing a special orientation, so that the $s$ and $p$ polarizations can be studied. The magnetic field polarization $p$ can be written in the following form:

1 $${H}_{1y}={\alpha }_{1}{e}^{i{\overrightarrow{k}}_{1}.\overrightarrow{r}}+{\beta }_{1}{e}^{i{\overrightarrow{k}}_{1}.\overrightarrow{r}}=\left({\alpha }_{1}{e}^{i{k}_{1z}z}+{\beta }_{1}{e}^{-i{k}_{1z}z}\right){e}^{-i{k}_{1x}x},\quad z<0$$

2 $${H}_{2y}={\alpha }_{2}{e}^{i{\overrightarrow{k}}_{2}.\overrightarrow{r}}+{\beta }_{2}{e}^{i{\overrightarrow{k}}_{2}.\overrightarrow{r}}=\left({\alpha }_{2}{e}^{i{k}_{2z}z}+{\beta }_{2}{e}^{-i{k}_{2z}z}\right){e}^{-i{k}_{2x}x},\quad z>0$$

Here, $c$ is the propagation speed of the light in space, $\omega$ is the angular frequency, ${\varepsilon }_{i} (i=1, 2)$ is the dielectric constant of the $i$ medium, ${\alpha }_{i}$ and ${\beta }_{i} (i=\mathrm{1,2})$ are the coefficients. $\vec{k}_{i} = \sqrt {\varepsilon_{i} } \omega /c\;\left( {i = 1,2} \right)$ is the wave vector of the electromagnetic wave. The wavevectors do not change at the material interfaces in the $x$ direction, that is, the $x$ components of the wave vectors at the interface are equal (${k}_{1x}={k}_{2x}$). According to Snell's law, if this situation is applied for the magnetic field and electric field at the interfaces, the following equations are obtained:

3 $${\left.{\widehat{n}}_{s}\times \left({\overrightarrow{E}}_{2}-{\overrightarrow{E}}_{1}\right)\right|}_{z=0}=0$$

4 $${\left.{\widehat{n}}_{s}\times \left({\overrightarrow{H}}_{2}-{\overrightarrow{H}}_{1}\right)\right|}_{z=0}=\overrightarrow{J}$$

$\overrightarrow{J}$ is the surface current density and ${\widehat{n}}_{s}$ is the normal unit vector of the surface. According to Ohm's law, the following set of equations can be derived at $z$=0:

5 $$\frac{{k}_{1z}}{{\varepsilon }_{1}}\left({a}_{1}-{b}_{1}\right)-\frac{{k}_{2z}}{{\varepsilon }_{2}}\left({a}_{2}-{b}_{2}\right)=0$$

6 $$\left({a}_{1}+{b}_{1}\right)-\left({a}_{2}+{b}_{2}\right)={J}_{x}$$

7 $${J}_{x}={\left.\sigma {E}_{x}\right|}_{z=0}=\frac{\sigma {k}_{2z}}{{\varepsilon }_{0}{\varepsilon }_{2}\omega }\left({a}_{2}-{b}_{2}\right)$$

where ${\varepsilon }_{0}$ is the permittivity of space. By combining Eqs. (5), (6) and (7), the coefficients ${a}_{i}$ and ${b}_{i}$ ($i=1$) can be combined with the coefficients ${a}_{i+1}$ and ${b}_{i+1}$ with the help of the transition matrix ${M}_{i\to i+1}$:

8 $$\left(\genfrac{}{}{0pt}{}{{a}_{i}}{{b}_{i}}\right)={M}_{i\to i+1}\left(\genfrac{}{}{0pt}{}{{a}_{i+1}}{{b}_{i+1}}\right)$$

for $i=1$, the transition matrix ${M}_{i\to i+1}$ is reduced to ${M}_{1\to 2}$:

9 $${M}_{1\to 2}=\frac{1}{2}\left(\begin{array}{cc}1+{n}_{p}+{\xi }_{p}\quad 1-{n}_{p}-{\xi }_{p}\\ 1-{n}_{p}+{\xi }_{p}\quad 1+{n}_{p}-{\xi }_{p}\end{array}\right)$$

where ${n}_{p}=\frac{{\varepsilon }_{1}{k}_{2z}}{{\varepsilon }_{2}{k}_{1z}}$, and ${\xi }_{p}=\frac{\sigma {k}_{2z}}{{\varepsilon }_{0}{\varepsilon }_{2}\omega }$. With the same methodology, the transition matrix for the s polarization is obtained as follows:

10 $${M}_{1\to 2}=\frac{1}{2}\left(\begin{array}{cc}1+{n}_{s}+{\xi }_{s}\quad 1-{n}_{s}+{\xi }_{s}\\ 1-{n}_{s}-{\xi }_{s}\quad 1+{n}_{s}-{\xi }_{s}\end{array}\right)$$

where ${\mu }_{0}$ is the magnetic permeability of space. The expressions ${n}_{s}$ and ${\xi }_{s}$ are equal to $\frac{{k}_{2z}}{{k}_{1z}}$ and $\frac{\sigma {\mu }_{0}\omega }{{k}_{1z}}$ respectively. ${n}_{p}$ and ${n}_{s}$ contain the refractive index of the x and y layers. In addition, the complex term includes the absorption coefficient. These values show a direct dependence on the wavelength and the angle of incidence of the electromagnetic wave. Boundary conditions and polarization states can be rewritten in the calculation of optical spectra depending on the angle of incidence of the electromagnetic wave.

All non-diagonal components of the transition matrices show similarity apart from a significant difference, for both s and p polarization. In this way, a common transition matrix for $j=(s,p)$ and ${\eta }_{p}=1$,${\eta }_{s}=-1$ can be arranged:

11 $${M}_{1\to 2}=\frac{1}{2}\left(\begin{array}{cc}1+{n}_{j}+{\xi }_{j}& 1-{n}_{j}-{{\eta }_{j}\xi }_{j}\\ 1-{n}_{j}+{{\eta }_{j}\xi }_{j}& 1+{n}_{j}-{\xi }_{j}\end{array}\right)$$

The transmission ($t$) and reflection ($r$) coefficients of the electromagnetic wave at the interfaces with the transition matrix and the transmittance ($T$), reflectance (R) and absorbance ($A$) spectra of the structure can be calculated as follows:

12 $${R= \left|r\right|}^{2}={\left|\frac{{M}_{2\to 1}}{{M}_{1\to 1}}\right|}^{2}$$

13 $${T= \left|t\right|}^{2}={\left|\frac{1}{{M}_{1\to 1}}\right|}^{2}$$

14 $$A=1-(T+R)$$

In the structure given in Fig. 2, when the thickness of the conductive layers is zero, the structure is reduced to the same structure as PC systems, which consist of periodically arranged structures with different refractive indices. In this case, the Bragg wavelength (${\lambda }_{B}$), which is the center wavelength corresponding to the resonance wavelength of the photonic band gap formed by the PC, must meet the following condition:

15 $$\frac{{\lambda }_{B}}{4}={n}_{i}{d}_{i}$$

where ${n}_{i}$ is the real part of the refractive index of each layer and ${d}_{i}$ is the layer thickness. For the same optical path in material with different refractive indices, when the ${n}_{i}{d}_{i}={n}_{j}{d}_{j}$ condition is met for the photonic crystal quarter-wave stack, the reflection takes its maximum value and coincides with the center of the 1^st^ order photonic band gap. Therefore, by determining the center of the photonic band gap to be designed, the thickness optimization of each layer in the photonic crystal is done with Eq. (15). As the angle of incidence of the electromagnetic wave increases, the ${\lambda }_{B}$ shifts to short-wavelengths and the bandwidth becomes narrower^25^.

The change in photovoltaic performances as a result of the integration of the 1D-PC, which is designed with the desired photonic band gap and properties, to the SC with CdS/CdTe heterojunction can be examined based on the absorption spectrum of the structure. In FTO/SnO_2_/CdTe/CdS/MoO_3_/(MgF_2_/MoO_3_)^N^ SC, the absorption characteristic and the wavelength dependent variation of AM 1.5G spectral irradiance (${S}_{AM1.5G}$) should be taken into account, significantly to determine how the period number of 1D-PC will change with the ${J}_{ph}$ that will occur in the cell. For ${J}_{ph}$, a calculation has to be made over the entire wavelength range of AM 1.5G spectral irradiance and the following equation is calculated^19,32,39^:

16 $${J}_{ph}=\underset{{\lambda }_{low}}{\overset{{\lambda }_{up}}{\int }}\frac{e\lambda }{hc}{S}_{AM1.5G}\left(\lambda \right) A\left(\lambda \right) d\lambda$$

where $e$ is the electron's charge and $h$ is Planck's constant.

### Experimental details

FTO/SnO_2_/CdS/CdTe/MoO_3_ SC and (MgF_2_/MoO_3_)^N^ 1D-PC system were deposited using RF sputtering method. Before placing the samples in the sputter system, the FTO-glass substrates were cleaned with alcohol, dried with pure nitrogen gas, and loaded into the Nanovak NVTS500 sputter system. SnO_2_, CdS, CdTe, MoO_3_, MgF_2_ targets were loaded on the substrates in the same medium, respectively. All targets used during coating have a diameter of 2", a thickness of 0.250" and a purity of 99.99%. All coatings were carried out in Ar plasma condition at room temperature and 20 mTorr pressure, with a power of 150 W and a rotational speed of 5 rpm. The distance between the target and the substrate holder is 100 mm. In order to provide thickness control and morphological stabilization, we kept the deposition rate as 0.04 Å/s.

With a suitable metal choice for CdTe, CdTe will form a Schottky barrier. Because CdTe has a very high electron affinity, a metal with a high work function is required to form an ohmic contact. Therefore, 300 nm thick Au metal contacts were made with the Bestec Thermal Evaporation System by masking in accordance with the design in Fig. 1.

To determine the optical characteristics of the produced SCs, transmittance measurements were taken with the Perkin Elmer Lambda 2S UV–Vis–NIR spectrometer in the 200–1100 nm range. In order to examine the photovoltaic performance of SCs, current density–voltage ($J-V$) characteristics were determined in the dark and AM 1.5G illumination. $J-V$ characteristics Keithley 4200 source meter and Newport Oriel-Sol1A solar simulator are used for AM 1.5G illumination. PCE and filling factor ($FF$) were calculated by determining the short-circuit current density (${J}_{sc}$), open-circuit voltage (${V}_{oc}$), maximum-current density (${J}_{m}$), and maximum voltage (${V}_{m}$) from the obtained $J-V$ curves.

## Data Availability

The datasets generated during and/or analysed during the current study are available from the corresponding author (Ç.Ç.) on reasonable request.
